# Risk Factors for Higher Postoperative Myocardial Injury in Minimally Invasive Mitral Valve Surgery Patients: A Cohort Study

**DOI:** 10.3390/jcm13061591

**Published:** 2024-03-10

**Authors:** Federica Baccanelli, Giovanni Albano, Alfonso Carrara, Matteo Parrinello, Claudio Roscitano, Maurizio Cecconi, Piersilvio Gerometta, Ascanio Graniero, Alfonso Agnino, Lorenzo Peluso

**Affiliations:** 1 Department of Anesthesia and Intensive Care, Humanitas Gavazzeni, Via M. Gavazzeni, 21, 24125 Bergamo, Italy; federica.baccanelli@gavazzeni.it (F.B.); giovanni.albano@gavazzeni.it (G.A.); alfonso.carrara@gavazzeni.it (A.C.); matteo.parrinello@gavazzeni.it (M.P.); claudio.roscitano@gavazzeni.it (C.R.); 2 Department of Biomedical Sciences, Humanitas University, Via Rita Levi Montalcini, 4, Pieve Emanuele, 20072 Milan, Italy; maurizio.cecconi@hunimed.eu; 3 Department of Anesthesia and Intensive Care, IRCCS Humanitas Research Hospital, Via Manzoni, 56, Rozzano, 20089 Milan, Italy; 4 Department of Cardiac Surgery, Humanitas Gavazzeni, Via M. Gavazzeni, 21, 24125 Bergamo, Italy; piersilvio.gerometta@gavazzeni.it (P.G.); ascanio.graniero@gavazzeni.it (A.G.); alfonso.agnino@gavazzeni.it (A.A.)

**Keywords:** troponin, cardiac surgery, robotic-assisted mitral valve surgery, cardiac anesthesia

## Abstract

**Background**: Postoperative myocardial injury, as detected by an elevated concentration of high-sensitivity cardiac troponin I (hs-cTnI), is a common complication in cardiac surgery that may be linked to mortality. The primary aim of this study was to assess the risk factors associated with increased myocardial injury in patients undergoing minimally invasive mitral valve surgery. **Methods**: In this retrospective monocentric cohort study, we analyzed all patients who underwent elective minimally invasive mitral valve surgery between January 2019 and December 2022 and were subsequently admitted to our intensive care unit. The study population was divided into two groups based on the peak hs-cTnI level: the “lower myocardial injury” group comprised patients whose peak serum hs-cTnI level was less than 499 times the 99th percentile, while the “higher myocardial injury” group included those patients who exhibited hs-cTnI levels equal to or greater than 500 times the 99th percentile. A multivariable logistic regression analysis was performed to identify independent risk factors associated with higher myocardial injury. **Results**: In our final analysis, we enrolled 316 patients. Patients with higher myocardial injury (48; 15%) more frequently had a preoperative New York Heart Association (NYHA) class ≥3 compared to those with lower myocardial injury [33 (69%) vs. 128 (48%); *p* < 0.01—OR 2.41 (95% CI 1.24–4.64); *p* < 0.01]. Furthermore, cardiopulmonary bypass and aortic cross-clamp time were significantly longer in the higher myocardial injury group compared to the lower myocardial injury group [117 (91–145) vs. 86 (74–100) min; *p* < 0.01—OR 1.05 (95% CI 1.03–1.06); *p* < 0.01]. Moreover, patients who underwent robotic-assisted mitral valve surgery experienced lower myocardial injury rates [9 (19%) vs. 102 (38%); *p* = 0.01—OR 0.38 (95% CI 0.18–0.81); *p* = 0.01] than others. These findings remained consistent after adjustment in multivariate logistic regression. In terms of postoperative outcomes, patients with higher myocardial injury exhibited the highest lactate peak in the first 24 h, a higher incidence of postoperative acute kidney injury and a longer duration of mechanical ventilation. Although no patients died in either group, those with higher myocardial injury experienced a longer hospital length of stay. **Conclusions**: Higher myocardial injury is relatively common after minimally invasive mitral valve surgery. Prolonged aortic cross-clamp duration and higher NYHA class were independently associated with myocardial injury, while robotic-assisted mitral valve surgery was independently associated with lower postoperative myocardial injury.

## 1. Introduction

Despite new surgical innovations and continuous medical improvements, cardiac surgery remains associated with some degrees of complications and still generates significant stress on different organs due to myocardial depression, systemic inflammatory response, catecholamines release and surgical procedures as well [1]. These side effects can persist through the postoperative period and may cause damage; thus, close monitoring during the immediate postoperative period is crucial for diagnosing and managing possible life-threatening complications promptly. Postoperative myocardial injury has always been reported after any open-heart surgery, and its value was related to the outcome [2,3]. Consistently, recent data confirmed that in patients who underwent cardiac surgery other than coronary artery bypass graft (CABG), a hs-cTnI peak higher than 499 times the 99th percentile was associated with 30-day mortality [4]. Biologic markers for myocardial necrosis (creatine phosphokinase, myoglobin, cardiac troponin I and troponin T) are released by injured myocardium and several studies associated their blood level to perioperative outcomes after cardiac surgery. Globally, most hospitals use high-sensitivity cardiac troponin I (hs-cTnI) assays. Still, limited data are available to define a prognostically important degree of myocardial injury after cardiac surgery based on these laboratory results [4].

Mitral valve (MV) surgery remains poorly investigated, with the bulk of research predominantly centered on coronary artery bypass graft (CABG) surgery. Moreover, the existing body of literature primarily emphasizes the prognostic value of troponin in mortality prediction, leaving unresolved inquiries regarding potentially modifiable variables impacting the onset of postoperative myocardial injury subsequent to minimally invasive mitral valve surgery. Hence, the primary objective of our study is to delineate the independent risk factors associated with the emergence of postoperative myocardial injury.

## 2. Materials and Methods

### 2.1. Study Design and Patient Selection

This retrospective monocentric cohort study was performed in the Department of Anesthesia and Intensive Care at Humanitas Gavazzeni Hospital, Bergamo, Italy.

The study protocol was approved by the Local Ethical Committee (Prot. 05/24), and informed consent was waived because of the retrospective nature of the analysis.

From our institutional database, we selected all patients who underwent elective minimally invasive mitral valve surgery (i.e., valve repair or valve replacement), from January 2019 to December 2022.

The exclusion criteria were emergency or urgency surgery, combined MV surgery (i.e., MV and left appendage closure, MV and atrial fibrillation surgery, combined CABG or other valve surgeries) and patients with preoperative serum hs-cTnI above the gender-based 99° percentile.

Indications for intervention comprised moderate to severe MV regurgitation or stenosis at preoperative transesophageal echocardiography examination. In our institution, a heart team composed of a cardiac surgeon, anesthesiologist and cardiologist selected the patients and the indication for cardiac surgery or percutaneous approach (i.e., edge-to-edge repair with MitraClip© technology). Specifically, in our institution, a minimally invasive approach was taken for patients with surgical indication, which is the standard of care for isolated MV operation. The choice of performing a robotic approach versus a standard right thoracotomy was based on the surgical decision (i.e., anatomic characteristics and confidence of the surgeon with the technique).

### 2.2. Patients’ Care and Management

Preoperative patients evaluation included the following: laboratory tests (i.e., complete blood count, coagulation panel, basic metabolic panel, kidney, thyroid and liver function tests, cardiac biomarkers, C-reactive protein (CRP) and sexually transmitted infection tests), electrocardiography, transthoracic and transesophageal echocardiography, coronary angiography or CT coronary angiogram for younger patients, chest computed tomography (CT) scan and carotid ultrasound; moreover, specifically for minimally invasive mitral valve surgery, an abdominal CT scan for the study of abdominal aorta and femoral vessels for safe peripheral cannulation was performed. Elective patients were admitted to the hospital the day before surgery to complete the preoperative assessment.

Intraoperatively, all patients received general anesthesia with continuous infusion of propofol and repeated doses of fentanyl and rocuronium according to quantitative electroencephalography (qEEG); one-lung ventilation was necessary for robotic surgery, but not for non-robotic ones. The surgery was performed on cardiopulmonary bypass (CPB), and anticoagulation was achieved using intravenous unfractionated heparin given at a dose of 300 IU/kg with a target activated clotting time greater than 480 s. Weaning from CPB and the potential need of inotropic and vasopressors support was based on metabolic parameters and repeated transesophageal echocardiography performed by the anesthesiologist. All patients received 2 g of tranexamic acid during surgery, and, in case of bleeding problems, hemostatic treatment was thromboelastography-guided. Antimicrobial prophylaxis prescribed was cefazolin. All patients received Histidine-Tryptophan-Ketoglutarate solution as cardioplegia, and its amount was based on cross-clamp duration. After the surgery, all patients were admitted to the intensive care unit (ICU) for eventual hemodynamic stabilization and postoperative monitoring. Usually, after ICU admission, patients were rewarmed and hemodynamics were optimized based on transthoracic and/or transesophageal echocardiography. After carefully monitoring the blood loss and vital parameters, they were weaned from mechanical ventilation and were usually discharged after 48 h to the cardiac surgery ward. The surgical technique was the same in both groups and is described elsewhere [5,6].

### 2.3. Data Collection and Definitions

We collected data on demographics, comorbidities, EuroSCORE II, New York Heart Association (NYHA) class, indications and type of surgery, duration of CPB, aortic cross-clamp and operative time, lab tests, as well as ICU length of stay, blood loss and transfusion and major complications. We defined acute kidney injury (AKI) according to the current KDIGO guidelines [7]. In our ICU, serum hs-cTnI concentration is measured as part of a routine panel of blood test for each patient at least once per day. Hs-cTnI samples were analyzed using the chemiluminescent microparticle immunoassay (CMIA), the 99th percentile was 11.6 ng/L for women and 19.8 ng/L for men. The preoperative hs-cTnI value, the first three postoperative days and the highest hs-cTnI values were recorded for all patients.

We divided our study population into two groups based on the peak troponin level on the first postoperative day; specifically, the “lower myocardial injury” group was defined as having a peak serum hs-cTnI level less than 499 times the 99th percentile; conversely, the “higher myocardial injury” group was defined as having a peak serum hs-cTnI level was equal to or greater than 500 times the 99th percentile.

We decided to use this cut-off following the results of the VISION study, which showed that among patients who underwent cardiac surgeries other than CABG and aortic valve repair, this hs-cTnI value threshold was clinically relevant, with it being associated with an increased risk of death by 30 days.

### 2.4. Endpoint of the Study

The endpoint of this study was to assess the risk factors for higher myocardial injury in minimally invasive mitral valve surgery patients.

### 2.5. Statistical Analysis

Discrete variables were expressed as count (percentage) and continuous variables as mean ± standard deviation (SD) or median [25th–75th percentiles]. The Kolmogorov–Smirnov test was used, and histograms and normal-quantile plots were examined to verify the normality of the distribution of continuous variables. Demographics, clinical and differences between groups (higher myocardial injury vs. lower myocardial injury) were assessed using the chi-square test, Fisher’s exact test, Student’s *t*-test or Mann–Whitney U-test, as appropriate.

Multivariable logistic regression analysis with higher myocardial injury as the dependent variable was performed; collinearity between variables (i.e., a linear correlation coefficient higher than 0.3) was excluded before modeling; only variables associated with higher myocardial injury in the univariate analysis (*p* < 0.05) were included in the final model.

Odds ratios (ORs) with 95% confidence intervals (CIs) were computed using an enter model. We tested the fitness of the model using Hosmer and Lemeshow goodness-of-fit test.

All statistical tests were two-tailed, and a *p* value < 0.05 was considered statistically significant. Data were analyzed using IBM SPSS Statistics for Macintosh 25 (Armonk, NY, USA) and GraphPad PRISM version 8.0 (San Diego, CA, USA).

## 3. Results

### 3.1. Study Population

Of 349 patients who underwent MIMV surgery in the study period, we excluded 28 patients because they received combined surgery (i.e., mitral valve surgery and left appendage closure) and 5 patients for missing data on hs-cTnI levels. In our analysis, we finally enrolled 316 patients. The median age was 63 [53–71] years, and 118 (37%) of them were female. More common comorbidities were atrial fibrillation (19% of patients) and arterial hypertension (58% of patients), and just a minority of patients had diabetes, chronic arteriopathy, respiratory or neurologic disease. The median LVEF was 62 [60–66] %, and 161 (51%) patients belong to the NYHA class equal to or greater than 3. The median EuroSCORE II value of the study cohort was 0.92 [0.67–1.32]. One hundred and eleven patients (35%) received robotic-assisted mitral valve surgery; the median duration of CPB was 139 [118–167] min, and the median aortic cross-clamp time was 87 [75–104] min.

### 3.2. Higher Myocardial Injury versus Lower Myocardial Injury

**Figure 1:**
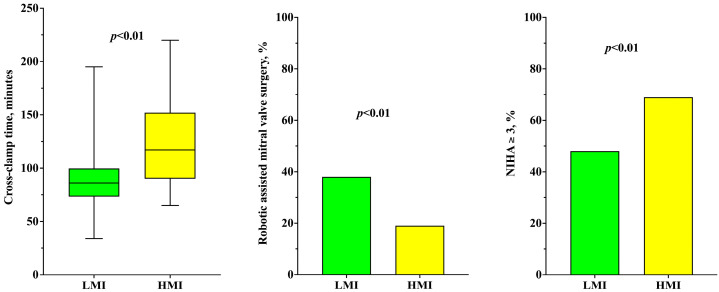
Timing of cross-clamp time (on the **left**), percentage of robotic-assisted mitral valve surgery (on the **center**) and percentage of patients with NYHA ≥ 3 (on the **right**), according to the development of high myocardial injury.

Patients’ preoperative characteristics such as age, sex, EuroSCORE II, comorbidities (AF, arterial hypertension, diabetes, COPD, neurologic diseases) and preoperative cardiac function (LVEF%) were similar in both higher and lower myocardial injury groups (Table 1). Conversely, patients with higher myocardial injury had preoperative NYHA class ≥ 3 more frequently than patients with lower myocardial injury [33 (69%) vs. 128 (48%); *p* < 0.01]. CPB (167 [134–209] vs. 135 [115–164] min; *p* < 0.01) and aortic cross-clamp time (117 [91–145] vs. 86 [74–100] min; *p* < 0.01—Figure 1) were significantly longer in the higher myocardial injury group.

Moreover, patients with higher myocardial injury underwent robotic-assisted mitral valve surgery less frequently [9 (19%) vs. 102 (38%); *p* = 0.01—Figure] and mitral valve replacement more frequently [6 (13%) vs. 10 (4%); *p* = 0.02] when compared to patients with lower myocardial injury.

At multivariate analysis (Table 2), aortic cross-clamp time (OR 1.05 [1.03–1.06]; *p* < 0.01) and a NYHA class ≥ 3 (OR 2.33 [1.04–5.22]; *p* = 0.04) were significantly associated with higher myocardial injury. Differently, robotic-assisted mitral valve surgery was found to be significantly associated with lower myocardial injury (OR 0.36 [0.14–0.90]; *p* = 0.03). Values of hs-cTnI over the first three days are shown in Table 3.

### 3.3. Postoperative Outcomes

Patients with higher myocardial injury showed highest lactate peak in the first 24 h (4.0 [2.6–6.7] vs. 2.7 [2.0–4.1] mmol/L; *p* < 0.01). Moreover, postoperative AKI (17% versus 5% of patients; *p* < 0.01) was significantly more frequent and the duration of mechanical ventilation (6 [4–11] vs. 5 [3–7] h; *p* < 0.01) was statistically longer in the higher myocardial injury group.

Finally, no patients died in either group, but higher myocardial injury patients showed a longer postoperative length of stay (10 [8–11] vs. 8 [7–10] days; *p* < 0.01) than those in the lower myocardial injury group (Table 1).

## 4. Discussion

In this retrospective cohort study, we showed that the duration of aortic cross-clamp and greater preoperative NYHA classes were independently associated with higher postoperative myocardial injury; conversely, robotic-assisted MV surgery was independently associated with lower postoperative myocardial injury.

In 2005, Khuri et al. had already highlighted that the occurrence of a 30-day postoperative complication was more important than preoperative patient risk and intraoperative factors in determining survival after major non-cardiac surgery [8].

Although cardiac surgery has the potential to improve the quality of life and increase survival, it remains a highly high-risk surgery, associated with severe and potentially life-threatening complications. Postoperative myocardial injury, detected by an elevated concentration of cardiac troponin, is one of the most common complications, associated with increased mortality [4].

Two preoperative risk scores are commonly used, the EuroSCORE II in Europe [9] and the Society of Thoracic Surgeons Score in the United States [10], to predict the risk of in-hospital mortality after cardiac surgery, but no biomarker-based risk score exists for the prediction of postoperative morbidity and mortality.

Troponin elevation occurs after every kind of cardiac surgery. This elevation may be procedural (e.g., incomplete myocardial protection, reperfusion injury, surgical trauma, defibrillation, etc.) or may reflect a postoperative onset of pathology (i.e., myocardial ischemia) or could be a combination of both mechanisms and, therefore, might become relevant for patient outcomes apart from initial risk stratification. Postoperative biomarker concentrations have been shown to independently and reliably improve prognostication in patients undergoing on-pump cardiac surgery [11,12].

It is well-established that the concentrations of cardiac troponin following cardiac operations are strongly predictive of postoperative adverse events, including cardiovascular instability, prolonged ICU length of stay, duration of mechanical ventilation, need for and the number of vasopressors, shock, postoperative impaired quality of life and both short and long-term mortality [13].

The mechanisms leading to myocardial damage after cardiac operations are multifactorial and also differ according to the type of surgery. In the literature, there is not a specific cut-off regarding hs-cTn to define acute postoperative myocardial damage in cardiac surgery, and several factors also complicate attempts to establish universally acceptable cut-off levels. Significant troponin elevations are expected after cardiac surgery, mostly in mitral valve operations. Still, these levels correlate poorly with clinically evident complications, so it remains challenging to interpret and use them to predict clinical outcomes and tailor patient care accordingly. Surgical techniques and patient case mix may vary greatly between institutions. The additional variation depends also on whether cTnI or cTnT is used, as well as the variety of different cTnI assays available. This means that the absolute values derived in any study cannot be universally applied.

The two most recent definitions and diagnostic criteria for perioperative myocardial injury/infarction stem from the Fourth Universal Definition of Myocardial Infarction (UDMI) [14] and the Academic Research Consortium-2 (ARC-2) [15] consensus documents. However, it is important to note that these criteria specifically address the first 48 h after coronary artery bypass graft (CABG) surgery. Both the UDMI and ARC-2 consensus statements acknowledge the arbitrary nature of their troponin thresholds. Additionally, there is a lack of consensus regarding which threshold may be clinically useful for cardiac surgery procedures other than CABG. Furthermore, most studies focusing on cardiac surgery procedures other than CABG have primarily utilized Troponin T [16,17]. It is widely recognized that high-sensitivity cardiac troponin assays offer greater precision and sensitivity compared to non-high-sensitivity assays [18].

To reduce the bias of absolute troponin values, we built a “weighted troponin” dividing the first-day peak troponin level with the 99th percentile, based on sex, that was provided by our laboratory. We have specifically chosen a cut-off of 499 times the 99th percentile as strongly associated with 30-day mortality, in accordance with previous literature 2. Interestingly, we found that postoperative myocardial injury was related to preoperative functional status (i.e., NYHA class) and intraoperative characteristics (i.e., aortic cross-clamp time and robotic-assisted technique).

Regarding the NYHA class, our results reinforce the importance of preoperative patients’ assessment and treatment as worse symptoms are invariably associated with worse outcomes [19]. Aortic cross-clamp duration has already been associated with postoperative myocardial damage even if few studies have tried to evaluate whether there is a “safe” cross-clamp time [20]. Immediately after the aortic cross-clamp, a cardioplegic solution is administered at different temperatures, concentrations and routes of administration; all these variables may have different effects on myocardial injury [21]. Moreover, when the administration of cardioplegia is suboptimal (i.e., retrograde administration, narrowed coronaries, aortic valve regurgitation) [22], or the interval between different administrations is excessive, the risk of reversible and irreversible cell damage is high.

Finally, to our knowledge, this is the first study showing an independent association between robotic-assisted MV surgery and a reduction in postoperative myocardial injury; this may be due to several factors. The association between robotic-assisted mitral valve surgery and shorter aortic cross-clamp time is controversial; apart from one study [23], most studies reported significantly longer times for robotic techniques [24,25,26,27,28,29,30]. We deem that the independent association with less myocardial injury might be related to the more precise and less traumatic heart incisions and manipulation.

Myocardial damage after MV operations remains inevitable after any on-pump cardiac surgery, and higher hs-cTnI levels are associated with 30-day mortality [4]. In our cohort, no patients died; we specifically assessed the in-hospital mortality, and our long-term results need to be confirmed. On the other hand, we observed a worsening in terms of prevalence of AKI, postoperative length of stay and duration of mechanical ventilation in the higher myocardial injury group; thus, we consider hs-cTnI an important marker of perioperative clinical outcomes.

Our study has several limitations. First, its retrospective design may have missed important confounding factors, and may not have completely adjusted for them. Second, being a monocentric study, its generalizability may be limited due to our clinical standard of practice. Third, the sample size is relatively small. Fourth, in postoperative echocardiography evaluation, we recorded only LVEF, and we did not specifically assess potential abnormalities in regional wall motion. Fifth, we did not report the long-term outcomes of our population.

## 5. Conclusions

In conclusion, our study highlights the prevalence of higher myocardial injury following on-pump minimally invasive mitral valve surgery. We identified prolonged aortic cross-clamp duration and preoperative higher NYHA class as independent risk factors for myocardial injury, while robotic-assisted mitral valve surgery appears to offer protective benefits. However, to elucidate and validate these findings, further prospective studies on a larger scale are warranted. Such investigations will provide deeper insights into the mechanisms and factors contributing to myocardial injury in this surgical context, ultimately guiding the optimization of patient care and outcomes.

## Figures and Tables

**Table 1:** Characteristics of the study population, according to the development of higher postoperative myocardial injury.

	Overall (*n* = 316)	Higher Myocardial Injury (*n* = 48)	Lower Myocardial Injury (*n* = 268)	*p* Values
Age, years	63 [53–71]	65 [56–73]	63 [52–71]	0.37
Female sex, *n* (%)	118 (37)	22 (46)	96 (36)	0.20
BMI	24.5 [22.1–26.6]	23.9 [22.5–25.8]	24.6 [22.1–26.6]	0.84
Preoperative Characteristics				
Atrial Fibrillation, *n* (%)	60 (19)	11 (23)	49 (18)	0.43
Mitral Valve Stenosis, *n* (%)	10 (3)	3 (6)	7 (3)	0.18
Arterial Hypertension, *n* (%)	183 (58)	32 (66)	151 (56)	0.21
Chronic Arteriopathy, *n* (%)	4 (1)	-	4 (2)	1.00
Diabetes, *n* (%)	15 (5)	3 (6)	12 (5)	0.71
Neurologic Disease, *n* (%)	29 (9)	3 (6)	26 (10)	0.59
Asthma/COPD, *n* (%)	26 (8)	4 (8)	22 (8)	1.00
Preoperative Hemoglobin	14.1 [13.1–15.0]	14.1 [13.2–14.9]	14.1 [13.0–15.0]	0.94
Preoperative Creatinine	0.90 [0.80–1.07]	0.86 [0.73–0.99]	0.91 [0.81–1.07]	0.10
EuroSCORE II, score	0.92 [0.67–1.32]	0.96 [0.63–2.19]	0.90 [0.67–1.25]	0.15
LV Ejection Fraction, (%)	62 [60–66]	60 [59–65]	62 [60–67]	0.33
NYHA class ≥ 3, *n* (%)	161 (51)	33 (69)	128 (48)	<0.01
Intraoperative Characteristics				
Robotic-Assisted Mitral Valve surgery, *n* (%)	111 (35)	9 (19)	102 (38)	0.01
Mitral Valve Replacement, *n* (%)	16 (5)	6 (13)	10 (4)	0.02
Cardiopulmonary bypass time, min	139 [118–167]	167 [134–209]	135 [115–164]	<0.01
Cross-clamp time, min	87 [75–104]	117 [91–145]	86 [74–100]	<0.01
Operative time, min	230 [200–265]	266 [218–313]	223 [200–256]	<0.01
IABP, *n* (%)	2 (1)	1 (2)	1 (1)	0.28
Postoperative Characteristics				
Bleeding at 24 h, mL	340 [250–495]	405 [265–540]	330 [250–480]	0.06
Highest Lactate at 24 h, mmol/L	2.9 [2.0–4.4]	4.0 [2.6–6.7]	2.7 [2.0–4.1]	<0.01
ICU Transfusions, *n* (%)	58 (18)	11 (23)	47 (18)	0.42
Surgical Revision, *n* (%)	12 (4)	3 (6)	9 (3)	0.40
Postoperative AF, *n* (%)	73 (23)	10 (21)	63 (24)	0.85
Postoperative AKI, *n* (%)	21 (7)	8 (17)	13 (5)	<0.01
Postoperative PCI, *n* (%)	2 (1)	2 (4)	-	0.02
LVEF at discharge, %	58 [55–60]	57 [55–60]	58 [54–60]	0.74
MV time, hours	5 [3–8]	6 [4–11]	5 [3–7]	<0.01
ICU stay, hours	44 [40–48]	45 [42–67]	44 [39–47]	0.06
Postoperative Length of Stay, days	9 [7–11]	10 [8–11]	8 [7–10]	<0.01
In-hospital Mortality, *n* (%)	-	-	-	-

Data are expressed as median [interquartile range] and count (percentage). Legend: BMI = Body Mass Index; COPD = Chronic Obstructive Pulmonary Disease; LV = Left Ventricular; NYHA = New York Heart Association; IABP = Intra-aortic Balloon Pump; ICU = intensive care unit; AKI = acute kidney injury; PCI = Percutaneous Coronary Intervention; MV = mechanical ventilation.

**Table 2:** Univariate and multivariate logistic regression analysis to higher postoperative myocardial injury.

	Univariate		Multivariate	
	Unadjusted OR [CI 95%]	*p* Value	Adjusted OR [CI 95%]	*p* Value
Age, years	1.01 [0.98–1.04]	0.33		
Female sex, *n* (%)	1.51 [0.81–2.82]	0.19		
BMI	1.00 [0.92–1.08]	0.99		
Atrial Fibrillation, *n* (%)	0.75 [0.36–1.58]	0.45		
Mitral Valve Stenosis, *n* (%)	0.40 [0.10–1.61]	0.20		
Arterial Hypertension, *n* (%)	0.65 [0.34–1.23]	0.18		
Diabetes, *n* (%)	0.70 [0.19–2.59]	0.60		
Neurologic Disease, *n* (%)	1.61 [0.47–5.55]	0.45		
Asthma/COPD, *n* (%)	0.98 [0.32–2.99]	0.98		
Preoperative Hemoglobin	1.02 [0.83–1.24]	0.88		
Preoperative Creatinine	0.37 [0.10–1.30]	0.12		
EuroSCORE II, score	1.38 [1.08–1.79]	0.01	1.22 [0.87–1.70]	0.24
LV Ejection Fraction, (%)	0.99 [0.94–1.04]	0.57		
NYHA class ≥ 3, *n* (%)	2.41 [1.24–4.64]	<0.01	2.33 [1.04–5.22]	0.04
Robotic-Assisted Mitral Valve surgery, *n* (%)	0.38 [0.18–0.81]	0.01	0.36 [0.14–0.90]	0.03
Mitral Valve Replacement, *n* (%)	3.86 [1.33–11.19]	0.01	2.36 [0.63–8.93]	0.21
Cardiopulmonary bypass time, min	1.02 [1.01–1.02]	<0.01		
Cross-clamp time, min	1.05 [1.03–1.06]	<0.01	1.05 [1.03–1.06]	<0.01
Operative time, min	1.02 [1.01–1.02]	<0.01		
IABP, *n* (%)	0.18 [0.01–2.86]	0.22		

Hosmer and Lemeshow goodness-of-fit: *p* = 0.80.

**Table 3:** High-sensitivity cardiac troponin I values over time, according to the development of higher postoperative myocardial injury.

	Overall (*n* = 316)	Higher Myocardial Injury (*n* = 48)	Lower Myocardial Injury (*n* = 268)	*p* Values
Troponin day 1, ng/L	4071 [2810–6518]	12,424 [7815–20,271]	3555 [2677–4918]	<0.01
Troponin day 2, ng/L	2124 [1472–3332]	6221 [3884–9311]	1872 [1347–2780]	<0.01
Troponin day 3, ng/L	1032 [687–1728]	3666 [1819–6200]	908 [640–1345]	<0.01

Data are expressed as median [interquartile range] and count (percentage).

## Data Availability

Data are available on request to the corresponding author.
